# EPICOVIDEHA: A Ready to Use Platform for Epidemiological Studies in Hematological Patients With COVID-19

**DOI:** 10.1097/HS9.0000000000000612

**Published:** 2021-06-25

**Authors:** Jon Salmanton-García, Alessandro Busca, Oliver A. Cornely, Paolo Corradini, Martin Hoenigl, Nikolai Klimko, Francesco Marchesi, Antonio Pagliuca, Francesco Passamonti, Philipp Koehler, Livio Pagano

**Affiliations:** 1 Department I of Internal Medicine, Faculty of Medicine and University Hospital Cologne, University of Cologne, Excellence Center for Medical Mycology (ECMM), Cologne, Germany; 2 Faculty of Medicine and University Hospital Cologne, University of Cologne, Chair Translational Research, Cologne Excellence Cluster on Cellular Stress Responses in Aging-Associated Diseases (CECAD), Cologne, Germany; 3 Stem Cell Transplant Center, AOU Citta’ della Salute e della Scienza, Turin, Italy; 4 Faculty of Medicine and University Hospital Cologne, Clinical Trials Centre Cologne (ZKS Köln), University of Cologne, Germany; 5 Faculty of Medicine and University Hospital Cologne, Center for Molecular Medicine Cologne (CMMC), University of Cologne, Germany; 6 German Centre for Infection Research (DZIF), Partner Site Bonn-Cologne, Cologne, Germany; 7 University of Milan & Fondazione IRCCS Istituto Nazionale dei Tumori, Milan, Italy; 8 Division of Infectious Diseases and Global Public Health, Department of Medicine, University of California San Diego, CA, USA; 9 Clinical and Translational Fungal-Working Group, University of California San Diego, La Jolla, CA, USA; 10 Section of Infectious Diseases and Tropical Medicine, Department of Internal Medicine, Medical University of Graz, Austria; 11 Department of Clinical Mycology, Allergy and Immunology, North Western State Medical University, St Petersburg, Russia; 12 Hematology and Stem Cell Transplant Unit, IRCCS Regina Elena National Cancer Institute, Rome, Italy; 13 Department of Hematological Medicine, Kings College Hospital NHS Foundation Trust, London, United Kingdom; 14 Department of Medicine and Surgery, University of Insubria and ASST Sette Laghi, Ospedale di Circolo of Varese, Italy; 15 Hematology, Fondazione Policlinico Universitario Agostino Gemelli - IRCCS, Rome, Italy; 16 Hematology, Università Cattolica del Sacro Cuore, Rome, Italy

Patients with hematological malignancies (HMs) are at a continuous risk for immunodeficiency, related either to the own malignancy or to the associated therapies, such as antineoplastic drug administration or hematopoietic stem-cell transplantation (HSCT). Baseline immunosuppression can be the main predisposing condition for the development of infectious diseases caused by different pathogens, such as bacteria,^1,2^ fungi,^3,4^ or viruses.^5–8^

At the end of December 2019, in the city of Wuhan, People’s Republic of China, a pneumonia caused by severe acute respiratory syndrome coronavirus 2 (SARS-CoV-2) was first described.^9^ As the situation rapidly evolved the coronavirus disease 2019 (COVID-19) pandemic commenced.^10^ One year after the first case, in April 2021, >130 million cases of COVID-19 were reported, of which 2.8 million died.^11^ HMs have been found to be prevalent in 2% of the COVID-19 patients in general.^12^

Triggered by the COVID-19 pandemic, the EPICOVIDEHA registry was initiated in February 2020 by members of the Scientific Working Group (SWG) Infection in Hematology of the European Hematology Association (EHA). In this project, we implement a cooperation platform between interested hematology departments from different hospitals, to assess epidemiological data on incidence and outcome of HM patients infected by SARS-CoV-2.

The main objective of EPICOVIDEHA is to describe the epidemiology and outcome of patients with HM and COVID-19. The secondary objectives are (1) to define the incidence and type of COVID-19 in HM patients (eg, asymptomatic, symptomatic, and severe); (2) to determine the admission into intensive care units (ICUs); (3) to estimate the frequency of pre-existing comorbidities; (4) to evaluate the acute mortality rate, within 30 days from diagnosis of COVID-19; (5) to analyze the rate of overall case-fatality; (6) to monitor the spatial-geographical patterns of the disease; and (7) to stratify patients per off-/on-therapy and the type of therapy (antineoplastic, immunosuppressive, corticosteroid, antibody and small molecule administration, chimeric antigen receptor T [CAR-T] cells application, or allogeneic/autologous HSCT performance).

We herein describe how EPICOVIDEHA is set up, maintained, ready to use, and open for contributions to explore different parameters of HM patients with COVID-19.

## Registry overview

**Figure 1.**
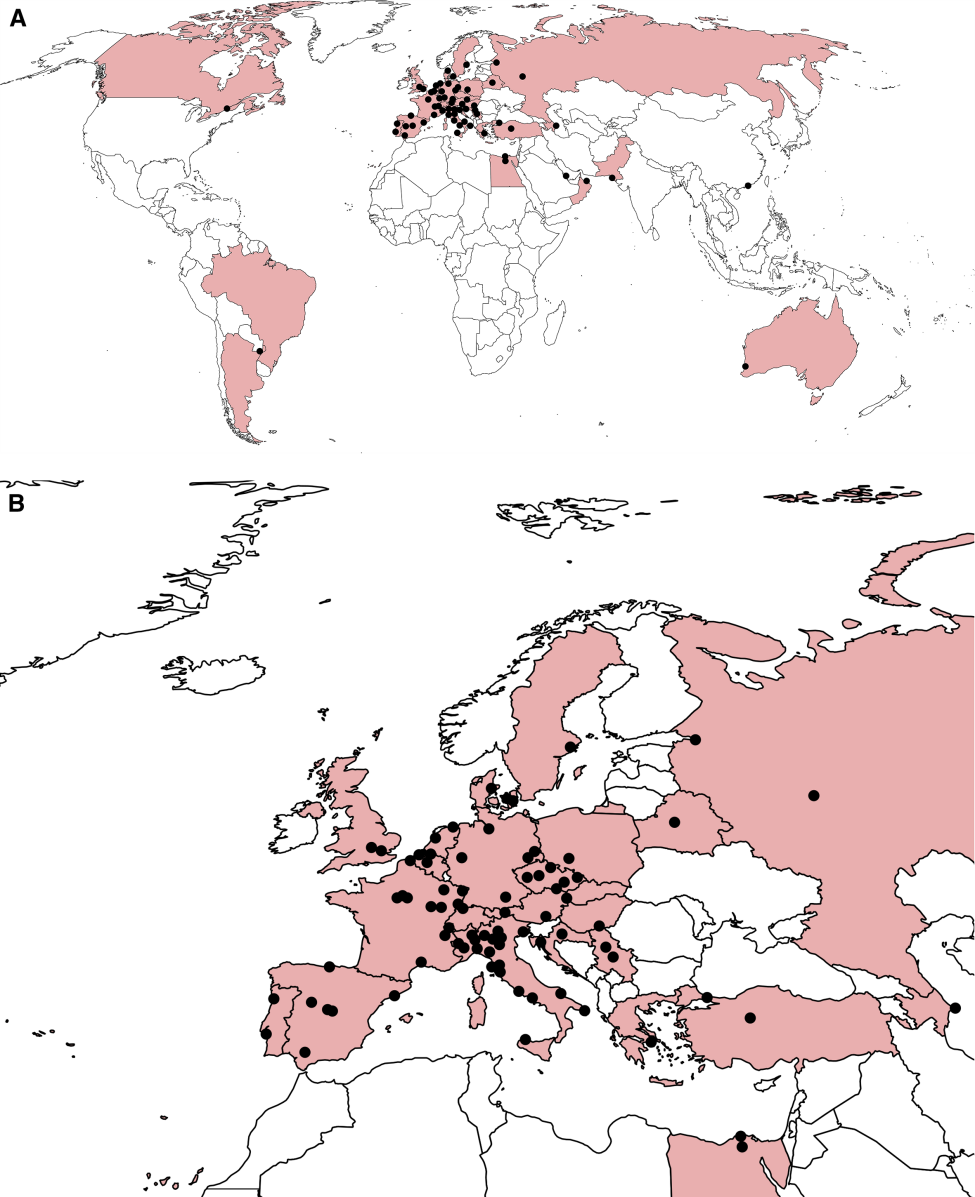
**Countries contributing to EPICOVIDEHA, as of April 2021.** (A), Countries colored in the map are: Argentina, Austria, Azerbaijan, Belarus, Belgium, Brazil, Canada, Croatia, Czech Republic, Denmark, Egypt, France, Germany, Greece, Hong Kong SAR, Hungary, Italy, The Netherlands, Oman, Pakistan, Portugal, Qatar, Russia, Serbia, Spain, Sweden, Switzerland, Turkey, and The United Kingdom. Name of institutions is listed in Supplemental Digital Table 1, http://links.lww.com/HS/A171. (B), European countries contributing to EPICOVIDEHA, as of April 2021. Countries colored in the map are: Austria, Azerbaijan, Belarus, Belgium, Croatia, Czech Republic, Denmark, France, Germany, Greece, Hungary, Italy, The Netherlands, Portugal, Russia, Serbia, Spain, Sweden, Switzerland, Turkey, and The United Kingdom. Name of institutions is listed in Supplemental Digital Table 1, http://links.lww.com/HS/A171.

EPICOVIDEHA is a multicenter retrospective, noninterventional, observational study. Researchers from different countries are invited to participate in the survey. In the initial phase, the participating centers will retrospectively review all episodes of COVID-19 disease occurring in patients with HM identified at their institutions from February 2020 onward. Then, every participating institution will be able to suggest further subanalyses, which will be approved by the steering committee of the project and performed during 2021. The steering committee of the project is composed of a panel of experts, mainly hematologists, with a long experience in the field of infectious complications in hematological patients representing 6 countries (AB, OAC, PC, MH, NK, AP, FP, PK, and LP). Currently, >120 institutions from 29 countries have registered >3500 cases in EPICOVIDEHA (Fig. 1).

### Case collection and documentation

EPICOVIDEHA seeks patients via different means. Initially, hematology medical societies (Arbeitsgemeinschaft Infektionen in der Hämatologie und Onkologie [AGIHO] of the Deutsche Gesellschaft für Hämatologie und Onkologie [DGHO], Czech Leukemia Study Group-for Life, Danish National Registry of COVID-19, EHA - SWG Infection in Hematology, Sociedad Española de Hematología y Hemoterapia [SEHH], Società Italiana di Ematologia [SIE], Sorveglianza Epidemiologica Infezioni nelle Emopatie [SEIFEM], and Supportive Treatment Group of the Croatian Cooperative Group for Hematological Diseases [KroHem]) have been contacted and informed about the survey. Additionally, close and long-term collaborators of the steering committee members and authors from related hematology publications have been approached. Moreover, the survey has been promoted in social media. Finally, researchers contributing cases in other surveys available at www.clinicalsurveys.net had also the option to enroll their patients in EPICOVIDEHA.

The EPICOVIDEHA electronic case report form (eCRF) is available at www.clinicalsurveys.net. This online survey is provided by EFS Fall 2018 (Questback, Cologne, Germany). The anonymized eCRF is the single access for each participating institution and is structured in different thematic pages, as follows: (1) identification; (2) demographics; (3) underlying diseases; (4) hematological malignancy; (5) COVID-19; and (6) outcome. The pages (2) demographics, (4) hematological malignancy, and (6) outcome, respectively, have specific subpages, where further details on the variables collected in the main pages are requested (Table 1).

**Table 1.** EPICOVIDEHA Registry—Information Categories Captured

Category	Subcategory
Identification	Institution, city, country, inclusion in other registries, already published
Demographics	Sex, age, date of birth, ethnic origin, date of COVID-19 diagnosis, strain of SARS-CoV-2, previous vaccination, and site of state during the COVID-19
Underlying diseases	Chronic cardiopathy (atrial fibrillation, hypertension, obstructive arteriopathy, etc.), chronic pulmonary disease (asthma, COPD, cystic fibrosis, fibrosis, etc.), diabetes (treated with insulin or antidiabetic oral drugs), liver disease, obesity (BMI > 30) or underweight (BMI < 18.5), renal impairment (creatinine > 2 mg/dl), smoking history, other risk factors, no risk factor identified, absolute leukocyte, neutrophil and lymphocyte number
Hematological malignancy	Type of malignancy, details on the diagnosis, state of the malignancy at COVID-19 diagnosis day, time span between malignancy and COVID-19 diagnosis, type of treatment (chemotherapy, radiotherapy, allogeneic HSCT, autologous HSCT, CAR-T, others, no treatment)
COVID-19	Identification method, reason for COVID-19 test, ICU stay during COVID-19 (invasive/noninvasive mechanical ventilation)
Outcome	Survival status at last contact, last day of follow up, date of death, overall and per ward hospital stay, reason for death

BMI = body mass index; CAR-T = chimeric antigen receptor T; COPD = chronic pulmonary obstructive disease; COVID-19 = coronavirus disease 2019; HSCT = hematopoietic stem-cell transplantation; ICU = intensive care unit; SARS-CoV-2 = severe acute respiratory syndrome coronavirus 2.

The inclusion criteria are (1) age equal to or greater than 18 years; (2) history of HM (eg, leukemias, chronic myeloproliferative disorder, lymphoma, myeloma, myelodysplastic syndrome, and myeloproliferative neoplasm) at any stage/status within the last 5 years before COVID-19; and (3) report of SARS-CoV-2 positive test (eg, bronchoalveolar lavage [BAL] and nasopharyngeal swab) documented by real-time reverse transcriptase polymerase chain reaction (RT-PCR) diagnostic panels. Patients (1) with hematological diseases other than HM or solid tumors; (2) with clinical COVID-19 diagnosis or not tested positive for SARS-CoV-2; and (3) patients off-therapy for longer than 5 years are not eligible.

### Quality control and analyses

Experts at the University Hospital Cologne, Cologne, Germany, with previous experience in the research and study of HM and infectious diseases, review each of the cases included in the registry individually for completeness and consistency. First, the information provided by the contributors is examined to ensure that all variables to be analyzed have been completed and no details are missing. Subsequently, the consistency of each patient’s data is determined. If there is incomplete, contradictory, or incoherent information, the collaborators are contacted to ask specific questions about each of their patients. If any open queries remain, the collaborators are approached to resolve remaining queries. As soon as there are no open queries left, the case is considered valid. A case can be declared invalid for various reasons, namely: (1) age under 18 years; (2) diagnosis of HM after COVID-19 onset; (3) double entry of the same case; (4) HM off-therapy or inactive for longer than 5 years before COVID-19 diagnosis; (5) incomplete data; (6) lack of positive SARS-CoV-2 test or COVID-19 clinical diagnosis; or (7) lack of underlying HM (hematological nonmalignant diseases—eg, hemolytic anemia, autoimmune thrombocytopenia, or solid tumors). If a case is declared invalid, it will be excluded from analyses.

Individual exports of the data will be performed for a general overview manuscript, as well as for dedicated topics proposed within the platform. All the results will be published in peer-reviewed journals. SPSS software will be employed for the statistical analyses (SPSS, version 25.0, Chicago, IL). Data will be summarized in frequencies and percentages, and median and interquartile range (IQR), as appropriate. Comparisons and regressions will be calculated according to the specific aims, needs, and requirements of the oncoming publications.

### Ethics and data protection

EPICOVIDEHA has been approved by the local Institutional Review Board and Ethics Committee of the Fondazione Policlinico Universitario Agostino Gemelli—IRCCS, Università Cattolica del Sacro Cuore of Rome, Italy (Study ID: 3226). The corresponding local ethics committee of each participating institution may approve additionally the EPICOVIDEHA study when applicable. EPICOVIDEHA is registered at http://www.clinicaltrials.gov, with the identifier (NCT number): NCT 04733729.^13^

## Outlook

EPICOVIDEHA is a ready-to-use online platform aiming to analyze and understand the epidemiology and outcome of HM patients developing COVID-19. In the current COVID-19 pandemic, it is of utmost relevance to rapidly develop of collaborative tools that enhance the quality of the scientific evidence. Registries procure controlled or uncontrolled level II evidence^14^ and are already in-use for the study of infectious diseases by international consortiums,^14,15^ including with COVID-19 patients.^16,17^

EPICOVIDEHA presents different incentives to the participating institutions reporting data to the platform. The first one is the inclusion of the participants as co-authors in the oncoming publications, based on weighted parameters, which will consider between others, the number of cases contributed to the respective work. In case a collaborator does not fulfill all the criteria to be mentioned as a co-author, they will be listed in a “collaborator” paragraph or in the acknowledgment section, after a comprehensive evaluation of their individual contribution to that particular publication. The second one is the option that EPICOVIDEHA brings to every participant to develop new studies within the umbrella of the platform, after a study plan is presented in front of the steering committee and approved by this.

This platform has certain limitations, apart from those directly related to the retrospective design of study. First, it might happen that not every eligible patient from the participating institutions is eventually reported. Due to the difficulties in critical care of patients during the pandemic, there might be lost patient records or insufficient data documentation which leads to exclusion during medical validation. Second, certain HM might be overrepresented compared to others due to the regional variability in the prevalence of malignancies. Last, most of the cases are likely to be from Europe. Despite these limitations, an international online registry offers to improve knowledge in the disease management and it is an easy and accessible tool for participants from any country.

Here, we present a ready-to-use platform focused on the collection of epidemiological data from patients with recent HM that have eventually developed COVID-19. This survey will contribute to a better understanding of the characteristics of these patients and to improved medical recommendations for their clinical management in general, including individualized patient clinical management protocols. The future trajectory of this pandemic remains uncertain, and hematological communities must continue to prepare for its widespread impact.

## Acknowledgments

The authors thank all contributors for their utmost contributions and support to the project during a pandemic situation and to Susann Blossfeld, Corinna Kramer, Laman Rahimli, and Ertan Sal for their administrative and technical assistance.

## Disclosures

AB had received lecture honoraria from Gilead Sciences, Merck, Pfizer Pharmaceuticals, Basilea, Biotest and Jazz Pharmaceuticals; he was a Board member of Gilead Science and Takeda. OAC was supported by the German Federal Ministry of Research and Education, was funded by the Deutsche Forschungsgemeinschaft (DFG, German Research Foundation) under Germany’s Excellence Strategy—CECAD, EXC 2030—390661388 and had received research grants from, is an advisor to, or received lecture honoraria from Actelion, Allecra Therapeutics, Al-Jazeera Pharmaceuticals, Amplyx, Astellas, Basilea, Biosys, Cidara, Da Volterra, Entasis, F2G, Gilead, Grupo Biotoscana, Immunic, IQVIA, Janssen, Matinas, Medicines Company, MedPace, Melinta Therapeutics, Menarini, Merck/MSD, Mylan, Nabriva, Noxxon, Octapharma, Paratek, Pfizer, PSI, Roche Diagnostics, Scynexis, and Shionogi. MH received research funding form Gilead, Pfizer, Astellas, Scynexis, and NIH. NK was a Board member of Gilead Science, MSD, Pfizer, and has been speaker for Gilead Sciences, MSD, Pfizer Pharmaceuticals, and Astellas Pharma, outside the submitted work. AP had received research funding from Gilead, Pfizer, and MSD. PK was supported by the German Federal Ministry of Research and Education and the State of North Rhine-Westphalia, Germany and had received nonfinancial scientific grants from Miltenyi Biotec GmbH, Bergisch Gladbach, Germany, and the Cologne Excellence Cluster on Cellular Stress Responses in Aging-Associated Diseases, University of Cologne, Cologne, Germany, and received lecture honoraria from and/or is advisor to Akademie für Infektionsmedizin e.V., Ambu GmbH, Astellas Pharma, European Confederation of Medical Mycology, Gilead Sciences, GPR Academy Ruesselsheim, MSD Sharp & Dohme GmbH, Noxxon N.V., and University Hospital, LMU Munich. LP was a Board member of Gilead Science, MSD, Pfizer, Basilea, Janssen, Novartis, Jazz Pharmaceutical, Cidara and has been speaker for Gilead Sciences, MSD, Pfizer Pharmaceuticals, Astellas Pharma, and a Consultant for Menarini. All the other authors have no conflicts of interest to disclose.

## Sources of funding

EPICOVIDEHA has received funds from Optics COMMIT (COVID-19 Unmet Medical Needs and Associated Research Extension) COVID-19 RFP program by GILEAD Science, United States (Project 2020-8223).

## Supplementary Material


