# Supplementary material for: EPICOVIDEHA: A Ready to Use Platform for Epidemiological Studies in Hematological Patients With COVID-19
Source: Hemasphere. 2021 Jun 25;5(7):e612. doi: 10.1097/HS9.0000000000000612 (PMC8232068; doi:10.1097/HS9.0000000000000612)
Supplement: Supplementary file 1 [file hs9-5-e612-s001.docx]

## Appendix

# EPICOVIDEHA – A ready to use platform for epidemiological studies in hematological patients with COVID-19

## Supplementary tables

**Supplementary table 1.** List of current participants as of April 2021

| **Country** | **City** | **Institution** |
| --- | --- | --- |
| Argentina | Posadas | Hospital Escuela de Agudos Doctor Ramón Madariaga |
| Austria | Graz | Medical University of Graz |
|  | Innsbruck | Medical University of Innsbruck |
| Azerbaijan | Baku | Azerbaijan Scientific Research Hematology and Transfusiology Institute |
| Belarus | Minsk | Minsk Scientific and Practical Center for Surgery, Transplantology and Hematology |
| Belgium | Brasschaat | Algemeen Ziekenhuis Klina |
|  | Brugge | Algemeen Ziekenhuis Sint Jan |
|  | Leuven | Katholieke Universiteit Leuven |
| Brazil | * | * |
| Canada | Montreal | McGill University Health Centre |
| Croatia | Rijeka | University Hospital Center Rijeka |
|  | Zagreb | University Hospital Center Zagreb |
|  |  | University Hospital Dubrava |
| Czech Republic | Brno | University Hospital Brno |
|  | Hradec Králové | University Hospital Hradec Králové |
|  | Olomouc | University Hospital Olomouc |
|  | Ostrava | University Hospital Ostrava |
|  | Pilsen | University Hospital Pilsen |
|  | Prague | Institute of Hematology and Blood Transfusion |
|  |  | University Hospital of Královské Vinohrady |
| Denmark | Copenhagen | Rigshospitalet |
|  | Roskilde | Zealand University Hospital |
| Egypt | Mansoura | Oncology Center, Mansoura University |
| France | Annecy | Centre Hospitalier Annecy |
|  | Besançon | Centre Hospitalier Universitaire de Besançon |
|  | Corbeil-Essonnes | Centre Hospitalier Sud Francilien |
|  | Dijon | Centre Hospitalier Dijon |
|  | Le Kremlin-Bicêtre | Hôpital Kremlin-Bicêtre, Assistance Publique – Hôpitaux de Paris |
|  | Montpellier | Centre Hospitalier Universitaire Montpellier |
|  | Mulhouse | Centre Hospitalier Mulhouse |
|  | Nancy | Centre d'Oncologie de Gentilly |
|  |  | Centre Hospitalier Universitaire de Nancy |
|  | Paris | Hôpital Cochin, Assistance Publique – Hôpitaux de Paris |
|  |  | Hôpital Pitié-Salpêtrière, Assistance Publique – Hôpitaux de Paris |
|  |  | Hôpital Saint Antoine, Assistance Publique – Hôpitaux de Paris |
|  |  | Hôpital Saint Louis, Assistance Publique – Hôpitaux de Paris |
|  |  | Necker-Enfants Malades Hospital, Assistance Publique – Hôpitaux de Paris |
|  | Saint-Cloud | Institut Curie |
|  | Strasbourg | ICANS - Institut de Cancérologie Strasbourg Europe |
|  | Versailles | Centre Hospitalier de Versailles |
|  | Villejuif | Institut Gustave Roussy |
| Germany | Cologne | University Hospital Cologne |
|  | Magdeburg | University Hospital Magdeburg |
| Greece | Athens | Attikon University General Hospital |
| Hong Kong SAR | Hong Kong | Pamela Youde Nethersole Eastern Hospital |
| Hungary | Szeged | South Division Faculty of Medicine University of Szeged |
| Italy | Barletta | Ospedale Dimiccoli Barletta |
|  | Brescia | Azienda Socio Sanitaria Territoriale degli Spedali Civili di Brescia Piazzale Spedali Civili |
|  | Genoa | Istituto Nazionale per la Ricerca sul Cancro Ospedale Policlinico San Martino |
|  | Lecce | Ospedale Vito Fazzi |
|  | Livorno | Azienda Toscana Nord Ovest |
|  | Lucca | Azienda Toscana Nord Ovest |
|  | Milan | Azienda Socio Sanitaria Territoriale Grande Ospedale Metropolitano Niguarda |
|  |  | Fondazione Istituto Nazionale per la Ricerca sul Cancro Ca' Granda Ospedale Maggiore Policlinico |
|  |  | Fondazione Istituto Nazionale per la Ricerca sul Cancro Istituto Nazionale dei Tumori |
|  |  | Istituto Nazionale per la Ricerca sul Cancro Ospedale San Raffaele |
|  | Monza | Azienda Ospedaliera San Gerardo - Monza |
|  | Naples | Azienda Ospedaliera di Rilievo Nazionale "Antonio Cardarelli" |
|  | Orbassano | San Luigi Gonzaga Hospital - Orbassano |
|  | Palermo | Azienda Ospedaliera “Ospedali Riuniti Villa Sofia-Cervello” |
|  |  | Azienda Ospedaliera Universitaria Policlinico Paolo Giaccone |
|  | Parma | Azienda Ospedaliera Universitaria Parma |
|  | Pisa | Azienda Ospedaliera Universitaria Policlinico Pisana - Cisanello |
|  | Rome | Fondazione Policlinico Universitario Agostino Gemelli |
|  |  | Istituto Nazionale per la Ricerca sul Cancro Regina Elena National Cancer Institute |
|  |  | Policlinico Tor Vergata of Rome |
|  | Turin | Azienda Ospedaliera Universitaria Città della Salute e Scienza di Torino |
|  | Udine | Azienda Sanitaria Universitaria del Friuli Centrale |
|  | Varese | University Insubria |
|  | Verona | Policlinico Borgo Roma Verona |
|  | Viareggio | Azienda Toscana Nord Ovest |
|  | Vicenza | Ospedale San Bortolo |
|  | Voghera | Azienda Ospedaliera Nazionale Santi Antonio e Biagio e Cesare Arrigo |
| Netherlands | Amsterdam | Amsterdam University Medical Center, location Vrije Universiteit Medical Center |
|  | Goes | Admiraal de Ruijter Hospital |
|  | Groningen | University Medical Center Groningen |
| Oman | Muscat | Sultan Qaboos University Hospital |
| Pakistan | Karachi | Aga Khan University |
| Portugal | Lisbon | Portuguese Institute of Oncology |
|  | Porto | Centro Hospitalar e Universitário São João |
| Qatar | Doha | Hamad Medical Corporation, National Center for Cancer Care and Research |
| Russia | Saint-Petersburg | North-Western State Medical University named after Ilya Ilyich Mechnikov |
|  |  | Pavlov University |
| Serbia | Belgrade | Clinic of Hematology, Clinical Center of Serbia |
| Spain | Barcelona | Hospital Clinic de Barcelona |
|  |  | Vall d'Hebron University Hospital |
|  | Iruña-Pamplona | Complejo Hospitalario de Navarra |
|  | Madrid | Fundacion Jimenez-Díaz |
|  |  | Hospital General Universitario Gregorio Marañón |
|  |  | Hospital Universitario 12 de Octubre |
|  |  | La Paz University Hospital |
|  | Majadahonda | Hospital Universitario Puerta de Hierro |
|  | Móstoles | Hospital Rey Juan Carlos |
|  | Salamanca | Hospital Universitario de Salamanca |
|  | Santander | Hospital Universitario Marqués de Valdecilla |
|  | Seville | Hospital Univesitario Virgen del Rocío |
| Sweden | Stockholm | Karolinska University Hospital |
| Switzerland | Basel | Universitätsspital Basel |
|  | Geneva | University Hospital of Geneva |
| Turkey | Ankara | Ankara Oncology Training and Research Hospital |
|  |  | Ankara University |
| United Kingdom | London | King's College Hospital |
|  | Oxford | Oxford University Hospitals |

* Cases from Brazil have been centrally collected by the Federal University of Rio de Janeiro, Rio de Janeiro
